# A climate-driven and field data-assimilated population dynamics model of sand flies

**DOI:** 10.1038/s41598-019-38994-w

**Published:** 2019-02-21

**Authors:** Kamil Erguler, Irene Pontiki, George Zittis, Yiannis Proestos, Vasiliki Christodoulou, Nikolaos Tsirigotakis, Maria Antoniou, Ozge Erisoz Kasap, Bulent Alten, Jos Lelieveld

**Affiliations:** 1 Energy, Environment and Water Research Center, The Cyprus Institute, 2121 Aglantzia, Nicosia Cyprus; 2 Laboratory of Clinical Bacteriology, Parasitology, Zoonoses and Geographical Medicine, School of Medicine, University of Crete, Heraklion, Crete Greece; 3 Faculty of Science, Department of Biology, Ecology Section, Hacettepe University, 06800 Beytepe-Ankara, Turkey; 4 Department of Atmospheric Chemistry, Max Planck Institute for Chemistry, D-55128 Mainz, Germany

## Abstract

Sand flies are responsible for the transmission of leishmaniasis, a neglected tropical disease claiming more than 50,000 lives annually. Leishmaniasis is an emerging health risk in tropical and Mediterranean countries as well as temperate regions in North America and Europe. There is an increasing demand for predicting population dynamics and spreading of sand flies to support management and control, yet phenotypic diversity and complex environmental dependence hamper model development. Here, we present the principles for developing predictive species-specific population dynamics models for important disease vectors. Based on these principles, we developed a sand fly population dynamics model with a generic structure where model parameters are inferred using a surveillance dataset collected from Greece and Cyprus. The model incorporates distinct life stages and explicit dependence on a carefully selected set of environmental variables. The model successfully replicates the observations and demonstrates high predictive capacity on the validation dataset from Turkey. The surveillance datasets inform about biological processes, even in the absence of laboratory experiments. Our findings suggest that the methodology can be applied to other vector species to predict abundance, control dispersion, and help to manage the global burden of vector-borne diseases.

## Introduction

Leishmaniasis is a neglected tropical disease caused by protozoan flagellates vectored by phlebotomine sand flies. The disease is associated with about 20 *Leishmania* species, and it manifests itself in various forms (the leishmaniases) including zoonotic and anthroponotic visceral and cutaneous leishmaniasis^1^. Primary vectors are *Phlebotomus* spp. in Eurasia and *Lutzomyia* app. in the Americas, and a diverse zoonotic reservoir including rodents and domestic dogs has been described^2^. Historically, leishmaniasis has been associated with poor living conditions and insufficient health-care^3^; however, crowding and poor urban planning in combination with warming trends in global climate are rapidly exacerbating conditions that lead sand flies and leishmaniasis towards temperate territories^4,5^.

The diseases caused by the *Leishmania* parasite are dynamic because sand flies are dependent on environmental, demographic and human behavioural factors. Such factors bring about changes in the habitat not only of the vectors but also of their natural hosts. At the same time, conditions resulting in immunosuppression in humans (like infection with human immunodeficiency virus (HIV) or organ transplantation-associated therapies) as well as the consequences of armed conflict, result in important changes in the epidemiology of the disease^6^. In order to safeguard public health, it is necessary to take account of sand fly and leishmaniasis risk factors.

Progress in mathematical modelling of vector-parasite systems is limited by the complex epidemiology and the availability of reliable surveillance data^7,8^. Nevertheless, known vector presence combined with climatic variables have been exploited for large-scale projections of habitat suitability. Fischer *et al*. performed environmental niche modelling with the maximum entropy methodology (MaxEnt) to model habitat suitability and project routes of dispersal across Central Europe driven by climate change^5^. Pigott *et al*. used boosted regression trees to infer environmental constraints and project global habitat suitability^2^. In a more recent study, Meneguzzi *et al*.^9^ performed environmental niche modelling to associate disease vectors with cutaneous leishmaniasis incidents in Southeastern Brazil^9^.

While environmental niche modelling provides a large-scale overview of possible environmental limitations of the vector species, mechanistic modelling with explicit links to the underlying biological processes offers a more detailed understanding of the environmental dependence of vector biology and disease epidemiology. Oshaghi *et al*. developed a population dynamics model of *Ph*. *papatasi* driven by local meteorological station data in Iran^10^. The authors used temperature thresholds from the literature^11^ to estimate accumulated degree days for life history processes, and adjusted laboratory derived parameters for field populations. The resulting correlation between predictions and observed population abundance suggests that laboratory-informed population dynamics modelling could be predictive provided that the parameters are properly adjusted for field conditions.

One of the key limitations of most population dynamics and disease transmission models, including the ones proposed for sand flies and leishmaniasis^12–16^, is their direct exploitation of laboratory experiments on key life-history traits, such as survival and development. Parameters derived from controlled experimental conditions may not be readily applicable for field populations especially while microclimate conditions around potential breeding sites cannot be reliably predicted with existing computational capacity and climate models. For these reasons, we recently proposed a Bayesian approach to develop population dynamics and disease transmission models by combining experimental data with field observations and observing the dynamics under the influence of large-scale environmental variables^17,18^. The emerging models demonstrated wide applicability and high predictive ability. However, the amount of experimental data on a vector or disease of interest is often limited due to the small number of experimental studies on model systems, the variety of vector and pathogen species, and the lack of host specificity^7^.

Here, we propose an adaptation of our Bayesian approach to assess the information content of field data obtained from multiple locations in informing predictive population dynamics models. We focus on three important vector species: *Phlebotomus neglectus*, *Phlebotomus tobbi* and *Phlebotomus papatasi*. While *Ph*. *neglectus* and *Ph*. *tobbi* are potent vectors of *Leishmania infantum*, which causes anthroponotic and zoonotic visceral leishmaniasis^19–21^, *Ph*. *papatasi* is a vector of *Leishmania major*, which causes zoonotic cutaneous leishmaniasis^22^. We developed a flexible and extensible framework for developing population dynamics models from field observations and performing climate-driven projections at a regional or global scale. We demonstrate that our approach is easily adjustable to study multiple vector species based on available surveillance data.

## Methods

**Figure 1:**
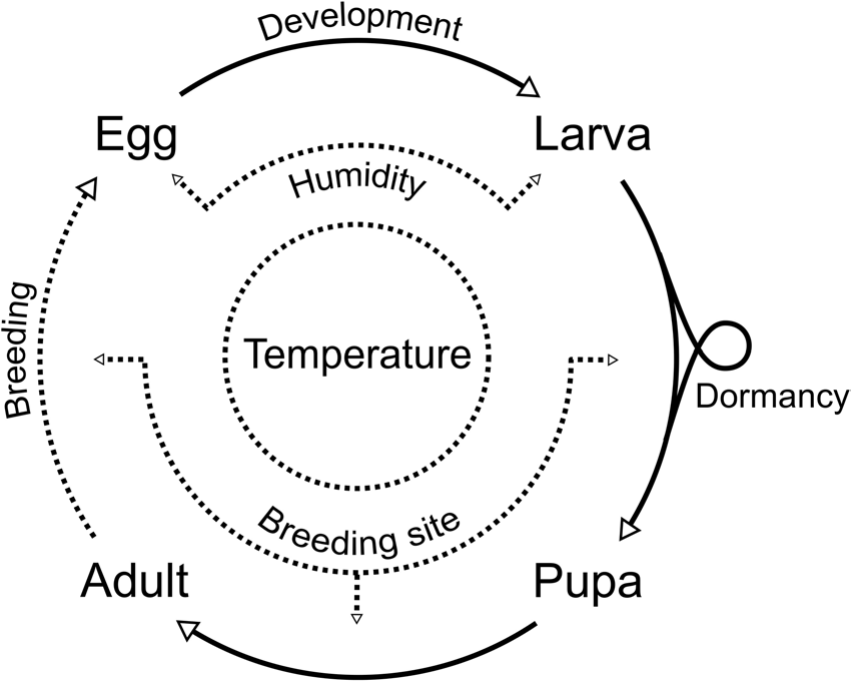
Flow diagram of the stochastic sand fly population dynamics model. The model is composed of four life stages including both male and female adult sand flies. Life stage parameters, such as survival, development, and fecundity are weather-driven, and a state of temperature-induced dormancy is assumed for the larval stage.

We developed a generic discrete-time stochastic model to represent the environmental dependence of sand fly populations. We present a schematic representation of the model in Fig. 1, and list model parameters in Table 1 together with domain boundaries used for parameter inference (see sec. Vector dynamics model). We implemented the model in ANSI C and integrated it with the albopictus Python package (v.1.6), which is available from the Python package index^23^.

**Table 1:** Model parameters with reference intervals.

Parameter	Definition	Prior interval	Notes
${n}_{E}(0)$, ${n}_{L}(0)$, ${n}_{P}(0)$, ${n}_{A}(0)$	Initial number of eggs, larvae, pupae, and adults, respectively	U (0,10^4^)^1^	
${d}_{{E}_{1}}$, ${d}_{{L}_{1}}$, ${d}_{{P}_{1}}$	Minimum mean development time of eggs, larvae, and pupae, respectively	U (1, 50) days	
${d}_{{E}_{2}}$, ${d}_{{L}_{2}}$, ${d}_{{P}_{2}}$	Maximum mean development time of eggs, larvae, and pupae, respectively	U (1, 50) days	Must be higher than the minimum
${d}_{{E}_{3}}$, ${d}_{{L}_{3}}$, ${d}_{{P}_{3}}$	Median developmental temperature for eggs, larvae, and pupae, respectively	U (−10, 50) °C	
${d}_{{A}_{1}}$	Minimum gonotrophic cycle duration	U (1, 50) days	
${d}_{{A}_{2}}$	Maximum gonotrophic cycle duration	U (1, 50) days	Must be higher than the minimum
${d}_{{A}_{3}}$	Median temperature for gonotrophic cycle duration	U (−10, 50) °C	
${p}_{{E}_{1}}$, ${p}_{{L}_{1}}$, ${p}_{{P}_{1}}$, ${p}_{{A}_{1}}$	Maximum daily survival probability of eggs, larvae, pupae, and adults respectively	U (0, 0.999)	
${p}_{{E}_{2}}$, ${p}_{{L}_{2}}$, ${p}_{{P}_{2}}$, ${p}_{{A}_{2}}$	Minimum survival temperature threshold for eggs, larvae, pupae, and adults respectively	U (−10, 50) °C	
${p}_{{E}_{3}}$, ${p}_{{L}_{3}}$, ${p}_{{P}_{3}}$, ${p}_{{A}_{3}}$	Maximum survival temperature threshold for eggs, larvae, pupae, and adults respectively	U (−10, 50) °C	Must be higher than the minimum
${F}_{{A}_{1}}$	Maximum number of eggs laid in a single gonotrophic cycle	U (0, 100)	
${F}_{{A}_{2}}$	Minimum temperature threshold for oviposition	U (−10, 50) °C	
${F}_{{A}_{3}}$	Maximum temperature threshold for oviposition	U (−10, 50) °C	Must be higher than the minimum
${p}_{g}$	Probability of surviving oviposition	U (0, 0.5)	
${p}_{x}$	Probability that a female adult emerges from a larva	U (0, 1)	
${p}_{\eta }$	Daily probability of getting caught	U (10^−4^, 10^−1^)	0.01–10% coverage is assumed
${{\rm{\Psi }}}_{T}$	Temperature threshold for dormancy	U (−10, 50) °C	
${{\rm{\Psi }}}_{H}$	Relative humidity threshold for egg and larva survival	U (0, 1)	
${{\rm{\Psi }}}_{B}$	Reduction in larva and pupa development probability and adult egg laying due to limited habitability of a breeding site	U (0, 1)	

### Vector dynamics model

We designed a stochastic difference equations model with Markov property, which implies that the state of a population on a given day is affected only by the state of the population and the environmental conditions on the previous day. We defined the population with the number of sand flies and the age structure in each stage. A single iteration comprises the cumulative effect of all stage transformations taking place during a day. Transformations include daily survival, development, egg laying, and, in case of an ongoing surveillance activity, being captured. For each transformation, we defined daily probabilities such as the daily probability of death or egg hatching.

Life history parameters of sand flies depend mainly on temperature, and, to a certain extent, on humidity^11,24,25^. In an attempt to standardise environmental dependency for the extensively diverse *Phlebotomus* genera, we employed single- or double-sided sigmoid curves relating temperature and humidity to survival and development. Sigmoid curves are frequently used in machine learning and fuzzy-logic classification where class boundaries are poorly defined^26^. Here, we employed sigmoid curves as generic approximations of environmental dependence where experimental observations are limited and observational error is large.

In order to limit complexity, we took account of the scarcity of experimental data, and assumed that temperature and humidity affect survival independently. We assumed that temperature strongly drives development and survival of all stages, and humidity is a requirement for the survival of the early immature stages, *i*.*e*. eggs and larvae. In essence, sufficient humidity is required for eggs and larvae to sustain viability^27^, and when humidity is sufficiently high, survival becomes dependent mainly on temperature. We used a single-sided sigmoid curve to describe egg survival as a function of relative humidity,

$${p}_{{{\rm{surv}}}_{{\rm{RH}}}}=1/(1+{e}^{\frac{{{\rm{\Psi }}}_{H}-{\rm{RH}}}{0.01}}),$$

where RH represents daily average relative humidity and ${{\rm{\Psi }}}_{H}$ represents the minimum RH for survival. The normalisation factor, 0.01, is to constrain the left and right tails of the curve within 0–1.

We used a double-sided sigmoid curve to describe temperature-driven survival. For instance, we described the daily probability of temperature-driven egg survival with

$${p}_{{{\rm{surv}}}_{T}}=\frac{{p}_{{E}_{1}}}{(1+{e}^{{p}_{{E}_{2}}-T})\,(1+{e}^{T-{p}_{{E}_{3}}})},$$

where *T* is daily average near-surface temperature (2 meters above ground). The parameters constraining the curve are defined in Table 1.

Survival probability changes daily as a result of temperature and humidity differences. In a hypothetical scenario where these variables are fixed, the survival process can be described with a geometric distribution with a constant probability $p=1-{p}_{{{\rm{surv}}}_{T}}$. In this case, the average life span can be calculated as (1 − *p*)/*p*.

We assumed that the development process is independent of survival, and modelled development time for eggs, larvae, pupae, and the gonotrophic cycle duration for adult females with a gamma distribution. Under constant environmental conditions, we assumed that each process lasts on average *μ* days with a standard deviation of *σ*. Together, *μ* and *σ* describe the probability of the duration of a development process. The resulting daily probability for the completion of development, *p*_dev_, can be written as

$${p}_{{\rm{dev}}}=\frac{{\rm{\Pr }}(d < x\le d+1)}{{\rm{\Pr }}(d < x)}$$

for day *d*, where $x\sim {\rm{\Gamma }}(\mu ,\sigma )$. In order to limit the total number of parameters for this generic model structure, we assumed that the variance of the gamma distribution is equal to its mean (${\sigma }^{2}=\mu$). We argue that this assumption is reasonable with regards to the experimental data (see sec. Experimental datasets); however, improvements and customisations are advisable on specific life history traits as more data become available.

Immature stage development and the gonotrophic cycle are hampered at extremely low temperatures^11,24^. In order to model these processes, we used a single-sided sigmoid curve,

1 $${d}_{{\rm{mean}}}={d}_{{\rm{\max }}}-\frac{{d}_{{\rm{\max }}}-{d}_{{\rm{\min }}}}{1+{e}^{{T}_{{\rm{med}}}-T}},$$

where *d*_min_ is the minimum, *d*_max_ is the maximum duration, and *T*_med_ is the temperature with the median duration. Equation 1 results in a mirrored S-shaped curve yielding long development times at low temperatures and a rapid development process at high temperatures.

Although female sand flies usually require blood feeding to develop eggs, egg laying and feeding patterns are discordant (egg laying can be delayed until after multiple blood meals) in certain species^19^, and reproduction is autogenous (egg laying requires no blood meal) in others^22^. Therefore, we assumed that the duration of the gonotrophic cycle is influenced mainly by temperature^24^. Mortality is strongly elevated after oviposition, where only a few females survive for the second, probably last, oviposition^28^. In order to model this unusually high death rate, we defined *p*_*g*_ as the probability of surviving egg laying for each female. At each gonotrophic cycle, a healthy female can lay up to 100 eggs^29^, which is observed to depend on temperature^24^. According to this, we used a double-sigmoid curve for the number of eggs laid,

$${p}_{{{\rm{FA}}}_{T}}=\frac{{F}_{{A}_{1}}}{(1+{e}^{{F}_{{A}_{2}}-T})\,(1+{e}^{T-{F}_{{A}_{3}}})},$$

where ${F}_{{A}_{1}}$ is maximum number of eggs a female can lay per cycle, and ${F}_{{A}_{2}}$ and ${F}_{{A}_{3}}$ are the minimum and maximum, respectively, temperatures for fecundity.

Aside from egg laying, we assumed that male and female sand flies share similar survival and developmental characteristics in all life stages^24,30^. In order to determine the number of fertile female adults, we defined *p*_*x*_ as the probability of emergence of a female adult sand fly from a developing pupa.

Facultative diapause, a mechanism of sustaining development in response to environmental factors, is reported in the 4^*th*^ instar larva in several species including *Ph*. *neglectus*, *Ph*. *tobbi*, and *Ph*. *papatasi*^10,19,31^. In order to accommodate the state of environmentally driven dormancy, we defined ${{\rm{\Psi }}}_{T}$ and updated the development time given in Eqn. 1. According to this, when temperature is lower than this threshold, $T < {{\rm{\Psi }}}_{T}$, larval development time is set to an extreme value (1000 days) to delay for as long as possible. Otherwise, development proceeds as given in Eqn. 1.

Sand fly abundance and diversity are affected strongly by the availability of appropriate breeding grounds. Although precipitation is not a direct influence, existence of rocky slopes, vegetation, availability of decaying organic matter, and an alkaline soil pH are known to contribute to abundance and diversity^32–34^. In order to capture breeding site conditions, we defined ${{\rm{\Psi }}}_{B}$ as a location-specific fraction, which reduces fecundity, prolongs egg laying interval, and extends the development times of larvae and pupae. According to this, ${{\rm{\Phi }}}_{B}=1$ indicates perfect breeding conditions where development and fecundity depend only on temperature and humidity. When ${{\rm{\Phi }}}_{B} < 1$, development rate is reduced due to adverse conditions such as limited availability of food or resting sites. We implemented this effect by increasing average development times (*μ* and *σ*) and reducing fecundity (${p}_{{{\rm{FA}}}_{T}}$) by a factor of ${{\rm{\Phi }}}_{B}$. We note that ${{\rm{\Psi }}}_{B}$ can only be inferred when performing inference on multiple locations. When a single location is in focus, ${{\rm{\Psi }}}_{B}$ merges with the rest of the parameters that drive development rate, *e*.*g*. ${p}_{{E}_{1}}$, ${p}_{{L}_{1}}$, ${p}_{{P}_{1}}$, and ${p}_{{A}_{1}}$.

The constant indicator implies that a breeding site provides sustained levels of availability and habitability. In addition, we assumed that the main drivers of population dynamics are climatic variables, and that population growth under the influence of these drivers would not reach high levels at which density or predation becomes a limiting factor. The effect of breeding site conditions on survival, therefore, is indirect as delayed development increases exposure to adverse weather conditions. In the current form of the model, ${{\rm{\Psi }}}_{B}$ is an arbitrary indicator and a first-order approximation of a complex and time-dependent phenomenon. Although this might be adequate for a generic model, it is subject of improvement in future work.

Although the flight range of adult sand flies is limited, they are known to be dispersed over long distances with the help of wind^5,27,31^. We postpone the discussion of the effect of migration and dispersion to future work where spatiotemporal modelling will be considered. In the present context, we assumed that migration and wind have negligible effects on the abundance of sand fly populations.

In addition to environmental constraints, we modelled the impact of surveillance activities on the abundance of adult sand flies. We defined ${p}_{\eta }$ as the daily probability of either a male or a female adult being captured and removed from the system. Accordingly, we defined the probability of an observation (*δ*) as the product of the binomial capture probabilities at each time point,

$$f(\delta |y)=\{\begin{array}{ll}\prod _{t,{\rm{sex}}}\,{\rm{Binom}}(y(t),\delta (t),{p}_{\eta }) & {\rm{for}}\,y(t)\ge \delta (t)\,\forall \,t\\ \,\,\,\,\,\,0 & {\rm{otherwise}}\end{array},$$

where *y*(*t*) is the predicted number of adult males or females, and *δ*(*t*) is the number of captured sand flies for day *t*. Predictions and observations comprise the number of female or male sand flies collected over three days as given in the surveillance procedure (see sec. Surveillance and environmental datasets below). In this equation, Binom(*n*, *m*, *p*) represents the binomial probability of choosing *m* sand flies out of a total of *n* with probability *p*.

We note that *y*(*t*) needs to be greater than or equal to *δ*(*t*) for all time points for the binomial probability to be defined. We handled the special case when this condition is not satisfied by replacing the binomial probability with an infinitesimal indicator value (<*e*^−13^) at problematic time points. The non-zero probability provides an indication for the degree of mismatch and a gradient to guide the optimisation routine towards more plausible parameter values. We note the fraction of times simulations yield such problematic time points and indicate the degree of success ($\rho$) throughout the text.

Likelihood of an observation in the context of Bayesian inference can be calculated as the weighted average of the binomial capture probability,

2 $$f(\delta |\theta )=\int \,f(\delta |y)f(y|\theta )dy,$$

where *y* is a prediction, *i*.*e*. a sample, from the model when its parameter values are given by *θ*. Due to limited computational resources, we approximated the likelihood with a Monte Carlo approximation,

3 $$f(\delta |\theta )\approx \frac{1}{N}\,\sum _{n=1}^{N}\,f(\delta |{y}_{n})f({y}_{n}|\theta ),$$

where *N* is the number of random samples from the model used to approximate the likelihood. More accurate approximations can be derived by using a larger sample size at the expense of computational resources; however, we found that *N* = 3 performs well in identifying an optimum fit (see Results and Discussion).

The model captures the essence of the environmental dependency of sand fly populations; however, we expect that species-specific differences, microclimate conditions around the breeding sites, day-night differences, intrinsic variability associated with the limited population size, and many other unaccounted factors may contribute to deviations between simulations and observations. Inevitably, such differences yield a very small likelihood value. In order to facilitate working with small values, we opted to work with the log-transformed likelihood, which we designated as the score,

4 $${\mathscr{S}}(\delta |\theta )=-\,\mathrm{ln}\,\frac{1}{3}\,\sum _{n=1}^{3}\,f(\delta |{y}_{n})f({y}_{n}|\theta ).$$

## Inference and posterior sampling

Having defined an objective function (Eqn. 4), we employed a uniform prior distribution as presented in Table 1 (*f*(*y*|*θ*) = *c*), performed parameter optimisation, and obtained posterior samples of *θ* using the python (v2.7) implementation of the hoppMCMC (v0.6) algorithm^17,18^. The hoppMCMC algorithm uses an adaptive Markov chain Monte Carlo (MCMC) method to obtain samples from the posterior distribution^35^.

For sufficiently large *N*, the Monte Carlo approximation (Eqn. 3) converges to the true likelihood, and the algorithm yields accurate posterior samples. When *N* is small, however, the error in the estimate, which is proportional to $1/\sqrt{N}$, may result in a prohibitively low acceptance rate. In order to compensate for small sample size, we performed posterior sampling with a tolerance. We scaled down the likelihood estimate exponentially with a constant, *C* < 1, to ensure that a minimum acceptance rate of 1% can be achieved. The constant corresponds to the inverse of the annealing temperature of the hoppMCMC algorithm. We report *C* where applicable. Details of inference and posterior sampling can be found in Supplementary File S1.

When reporting inference results, we adopted the concept of posterior modes as reported in previous work^17,18^. In essence, we assumed that the posterior distribution comprises a set of local maxima analogous to the local minima frequently encountered in optimisation. We assumed that the locality of each maximum leads to similar model behaviour, and the extent of each locality is dictated by the sensitivity around the maxima. In case of high sensitivity, only the parameter values in the vicinity of the maximum yield similar behaviour, and the behaviour changes drastically at greater distance. Although it is not trivial to sample from the entire posterior distribution, it is relatively straightforward to sample from the vicinity of a local maximum by starting a Markov chain from around that point^17^. Posterior samples obtained as such are labelled as samples from the same posterior mode. Throughout the text, we report posterior modes (single or multiple) as possible explanations for the observations.

## Experimental Datasets

**Figure 2:**
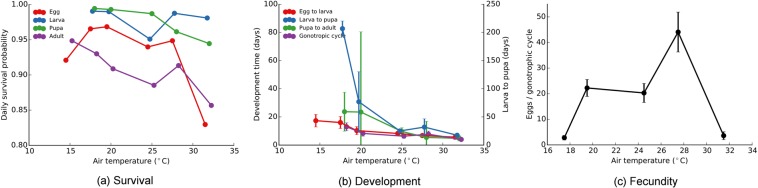
Life history parameters of *Ph*. *papatasi* derived under controlled environmental conditions. (**a**) Daily survival probabilities of eggs, larvae, pupae, and adults (both male and female). (**b**) Average development times of eggs, larvae, and pupae, and average gonotropic cycle lengths of adults. (**c**) Average number of eggs laid by an adult female at the end of each gonotropic cycle. Vertical ranges indicated in (**b**,**c**) correspond to the standard deviation.

Due to the cost and effort required to reliably determine life history parameters, comprehensive information on many sand fly species is yet to be obtained. Kasap *et al*. compiled a reliable data source on *Ph*. *papatasi* in two reports: Kasap *et al*.^11^ and Kasap *et al*.^24^. Here, we derived daily survival probabilities, development times, and fecundity for a range of temperatures using these data (Fig. 2).

We emphasise that these parameters originate from controlled environmental conditions, which include temperature, humidity, feeding, and photoperiod. Therefore, care must be taken when extrapolating them to be used with mathematical models due to pertaining structural restrictions (such as the existence of four distinct life stages) and simplifying assumptions (such as the use of daily average temperatures).

In this context, instead of using experimentally-derived values as informed prior probabilities, we reserved them for validation. By adopting this strategy, we were able to assess the information content of field observations and apply the same inference procedure to other *Phlebotomus* species for which have fewer experimental data.

We assumed that each observation is independent and normally distributed with the reported mean *d*_*T*_ and standard deviation *s*_*T*_, where *T* is the temperature. We estimated ${d}_{T}$ and ${s}_{T}$ empirically with the exception of ${s}_{T}$ for survival. We assumed ${s}_{T}=0.01$ for survival, which corresponds to 1% of the range (0–1). In order to assess the agreement between observed and inferred parameter values, we defined a score function,

$${\rm{\Pi }}(\theta )=\sum \,\frac{{[g(\theta ,T)-{d}_{T}]}^{2}}{2{s}_{T}^{2}},$$

where $g(\theta ,T)$ is the corresponding life history parameter indicated by the parameter configuration *θ* for temperature *T*. Consequently, the probability of *θ* given the experimental observations is proportional to ${e}^{-{\rm{\Pi }}(\theta )}$.

## Surveillance and environmental datasets

**Figure 3:**
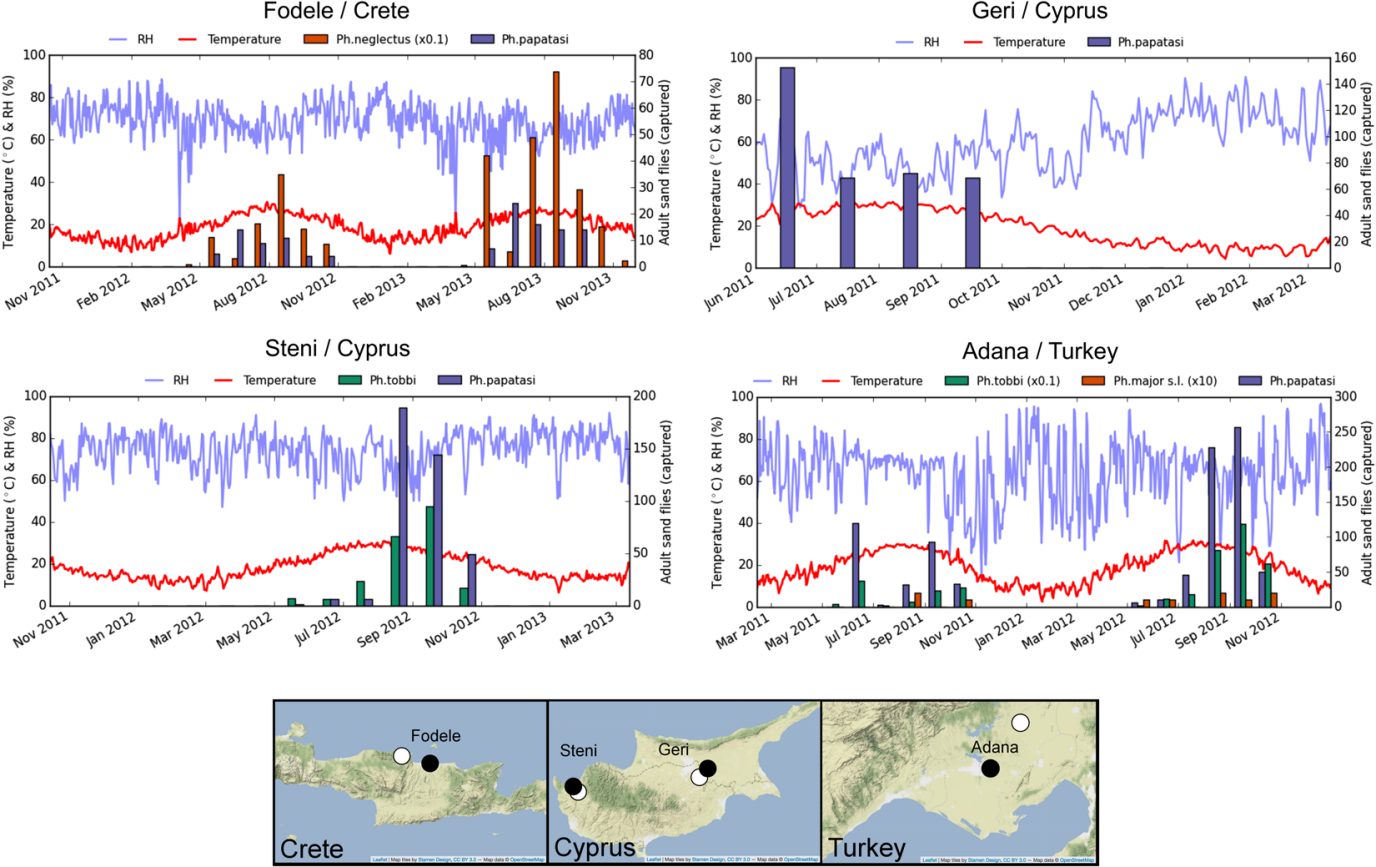
Surveillance and environmental datasets used in this study. Male and female sand fly counts from 2011 to 2013 together with the meteorological data (daily mean temperature and relative humidity) are plotted at the top. Coordinates of the traps are plotted as blue marks in the maps at the bottom. Green marks on the maps indicate the nearest weather stations from which environmental data are obtained. The maps were generated using the Leaflet (v1.1.0) library (http://leafletjs.com/) with the Stamen-TerrainBackground tiles to emphasise topography. Map tiles by Stamen Design (http://stamen.com, CC BY 3.0, http://creativecommons.org/licenses/by/3.0), and map data ©OpenStreetMap contributors (http://www.openstreetmap.org/copyright).

We allocated three surveillance datasets for parameter inference (training set) and one additional dataset for model validation (test set). The surveillance datasets comprise longitudinal abundance data from Greece (Fodele), Cyprus (Steni and Geri), and Turkey (Adana) collected as part of the comprehensive surveillance study reported in Alten *et al*.^36^. For each dataset, we obtained daily average near-surface temperature (2 meters above ground) and relative humidity from the open-access datasets of nearby meteorological stations. For Geri and Adana, we used the Integrated Surface Database Station History dataset of National Oceanic and Atmospheric Administration (NOAA)^37^. For Steni and Fodele, we used the blended meteorological datasets from the European Climate Assessment & Dataset (ECA&D)^38^. We list the collection sites and the sources of weather data in Table 2. In Fig. 3, we plot the abundance and weather data together for all the locations.

**Table 2:** Longitudinal datasets and corresponding weather stations.

Test/training	Collection site				Weather station		
	Country	Area	Longitude	Latitude	ID/dataset	Longitude	Latitude
Training	Greece	Fodele	24.958	35.381	Heraklion	25.183	35.333
Training	Cyprus	Steni	32.471	34.998	Polis	32.433	35.033
Training	Cyprus	Geri	32.471	34.998	Ercan_175210	33.500	35.150
Test	Turkey	Adana (Koyunevi)	35.655	37.289	İncirlik	35.417	37.000

In the training set, we included three patterns frequently encountered in surveillance studies: (i) An extended observational dataset in terms of temporal coverage, which follows two consecutive years in Fodele, (ii) a complete dataset, which follows the start and end of the sand fly high season in Steni, and an incomplete one, which starts late summer in Geri. We selected Adana for validation due to the similarity of its climate and land cover in spite of the distance and isolation with respect to the two islands.

As reported by Alten *et al*., data collection was carried out using a combination of light and sticky traps between April and November during three consecutive days (13–15^*th*^ days) in each month during 2011–2013^36^. Since sand fly presence is unlikely after November until the beginning of the next high season in April, no data was collected during this period. In order to constrain inference in the light of this expectation, we assumed zero presence for the unobserved months of the training set.

The surveillance data comprise counts of male and female adult *Ph*. *papatasi*, *Ph*. *neglectus*, and *Ph*. *tobbi*, which were identified morphologically. It is important to note that morphological identification of *Ph*. *neglectus*, which belongs to the *Ph. major s*.*l*. species complex, was not definitive in Adana as *Ph*. *neglectus* and *Ph*. *syriacus* of the complex share the same habitat in the region^39^. Nevertheless, in order to validate model predictions for *Ph. neglectus*, we used the combined abundance of the *Ph. major s*.*l*. complex as the closest available dataset.

## Results and Discussion

We assessed the inferability of a biologically-plausible environmentally driven population dynamics model using only sand fly surveillance data as the basis of inference. We assessed the predictive capacity of models inferred using various degrees of information, and presented guidelines for extending this procedure to other species.

### Multiple versions of the model are informed by the surveillance data

The sand fly model comprises a total of 37 parameters including 6 that are location-specific (the four initial conditions, the degree of habitability, and the daily probability of capture — see sec. Vector dynamics model). We inferred all of these using only a single location for each of the three species: *Ph*. *papatasi*, *Ph*. *neglectus*, and *Ph*. *tobbi* (see sec. Inference and posterior sampling). Then, we combined the three locations to infer all for *Ph*. *papatasi*, the only species found in all locations, while allowing three different values for the location-specific parameters.

As a result, when we used only one location for inference, we identified one major posterior mode for each. We identified multiple posterior modes when we combined all locations, and, here, we report three of the most representative and most probable ones labelled as “Combined A”, “Combined B”, and “Combined C”. In Table 3, we report the annealing temperature (*C*), the degree of success ($\rho$), the average score (${\mathscr{S}}(\delta |\theta )$), and the absolute value of the determinant of the covariance matrix (${\mathscr{U}}$). We report ${\mathscr{U}}$ as a measure of dispersion in posterior modes.

**Table 3:** Posterior modes obtained using the training datasets.

Species	Type	Area	*C*	$\rho$	${\mathscr{S}}$(*δ*|*θ*)	${\mathscr{U}}$
*Ph*. *neglectus*	Single	Fodele	4	100.0%	733.53 ± 19.12	79.6714
*Ph*. *tobbi*	Single	Steni	2	100.0%	44.29 ± 5.49	147.879
*Ph*. *papatasi*	Single	Geri	2	100.0%	31.36 ± 5.43	168.334
	Single	Steni	2	99.7%	41.71 ± 6.18	133.308
	Single	Fodele	2	100.0%	72.90 ± 5.51	138.493
	Combined A	Geri	2	100.0%	33.76 ± 3.85	104.499
		Steni		100.0%	54.61 ± 5.76	106.085
		Fodele		100.0%	96.11 ± 5.50	105.249
	Combined B	Geri	2	100.0%	33.40 ± 3.61	96.1643
		Steni		100.0%	47.82 ± 5.99	100.002
		Fodele		99.9%	92.73 ± 5.14	98.1116
	Combined C	Geri	2	100.0%	44.34 ± 5.17	103.678
		Steni		100.0%	134.80 ± 10.39	106.022
		Fodele		100.0%	85.51 ± 6.56	106.926

Single or combined inferences are listed with the associated sand fly species, annealing temperature (*C*), degree of success ($\rho$), average and standard deviation of the score (${\mathscr{S}}$(*δ*|*θ*)), and the extent of dispersion in posterior modes (${\mathscr{U}}$).

The likelihood function is proportional to the size of a dataset and the number of sand flies collected (Eqn. 2); therefore, a large number of collections or more data points yield high likelihood values. In turn, this constrains the extent of a posterior mode. In agreement with this, Fodele (the largest dataset) yields a less dispersed posterior mode than Geri (the smallest dataset). Combined inferences yield highly constrained posterior modes due to the cumulative size of the combined dataset. We observed the lowest dispersion for *Ph*. *neglectus* which was collected in large numbers. In order to preserve 1% acceptance rate in sampling, a higher annealing temperature was needed for this dataset.

**Figure 4:**
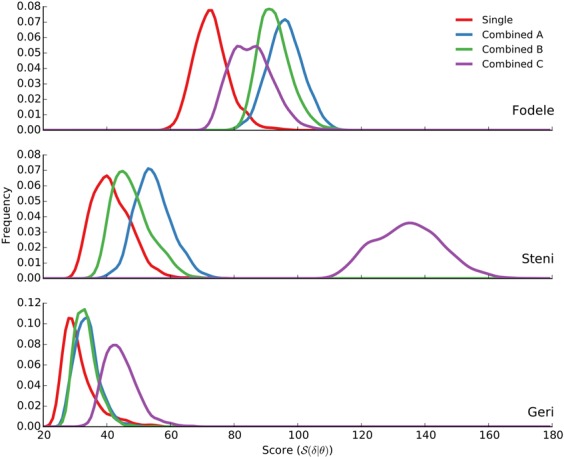
Score distributions of the posterior samples for *Ph*. *papatasi*. Kernel density estimates for samples from 6 posterior modes (3 single + 3 combined inferences) are shown for the three locations in the training set. Posterior modes from single inferences are different from each other, and yet, they are shown in red for simplicity. Blue: Combined A, green: Combined B, and purple: Combined C.

Estimates of kernel density of the scores associated with the inference of *Ph*. *papatasi* can be seen in Fig. 4. We found that single location inferences explain each location better than combined inferences. Posterior modes labelled “Combined A” and “Combined B” yield balanced fits to the three locations, while “Combined C” tends to fit Fodele better than the other two locations. Resulting matches with the data for all inferences are shown in Supplementary Fig. S1.

**Figure 5:**
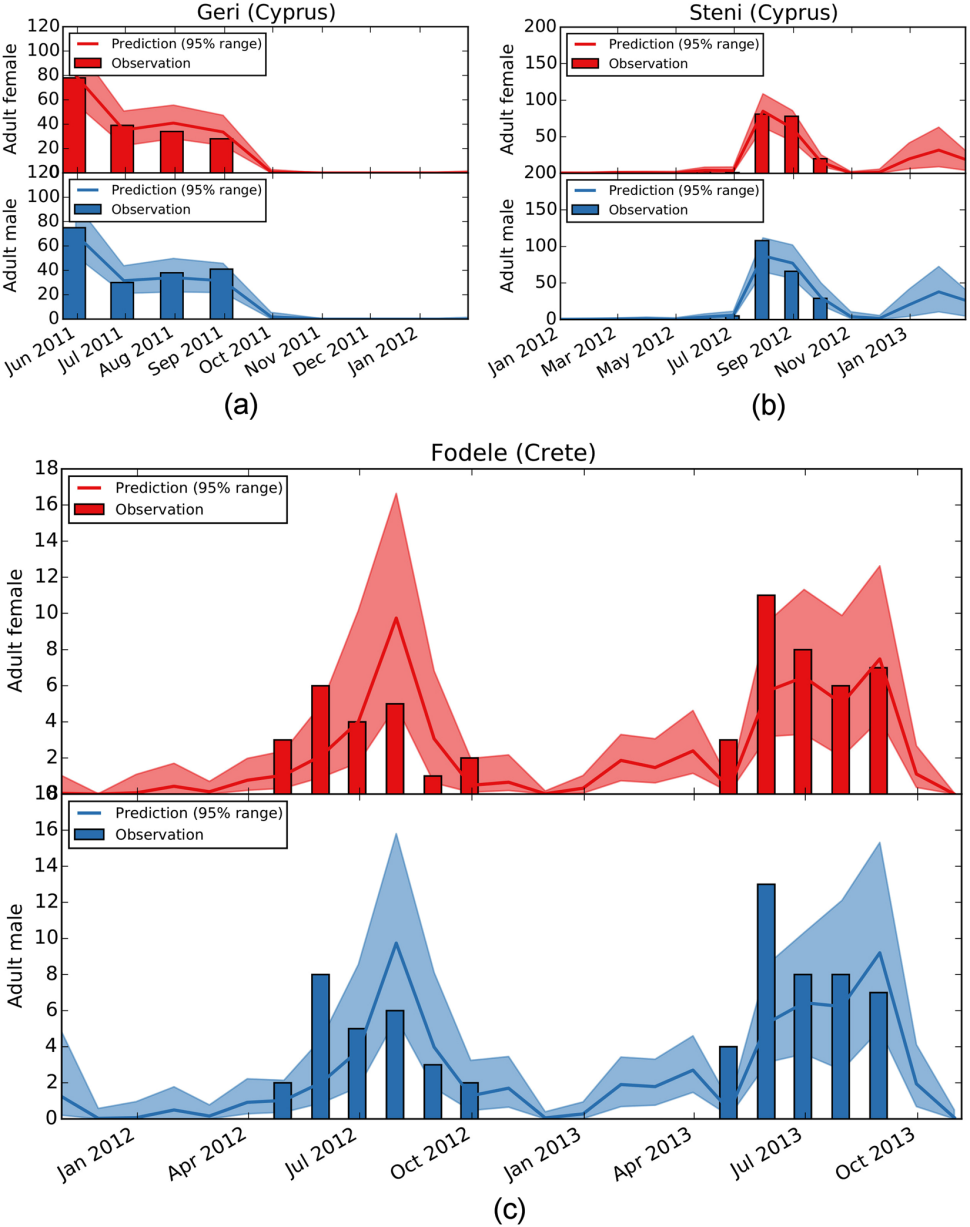
Agreement between *Ph*. *papatasi* surveillance data and model predictions with Combined A. Number of adult sand flies collected from Geri, Steni, and Fodele are plotted as bars in (**a**–**c**), respectively. Solid lines and shaded areas indicate the median and the 95% range of the predictions. Female and male adult sand flies are marked in red and blue, respectively.

In Fig. 5, we present the match between surveillance data and model predictions with Combined A. We note that the median predictions for winter, despite being explicitly set to zero in the training set, are relatively low, but rarely zero in Fodele. This suggests that a continuous, yet minimal, sand fly activity might be present in the region during winter. One of the consistent predictions by all posterior modes (Supplementary Fig. S1) is that the beginning of the sand fly high season may be several months earlier in Fodele than the beginning of the surveillance period (April).

An interesting pattern in the surveillance data from Fodele is a peak occurrence both in males and females in June in both years of observations. Neither the single-location inference nor the combined ones fully explain these peaks for both sexes. The mismatch is indicative of a biological mechanism not accounted for in this version of the model. This could be, for instance, an alternative type of dormancy, such as obligate diapause, where termination is spontaneous and independent of external stimuli. Seasonal changes in breeding sites or feeding patterns could also result in the observed peaks. The understanding of this mechanism requires further iterations of the model, which will be the subject of continued research.

### Surveillance data sufficiently informs predictive models

**Figure 6:**
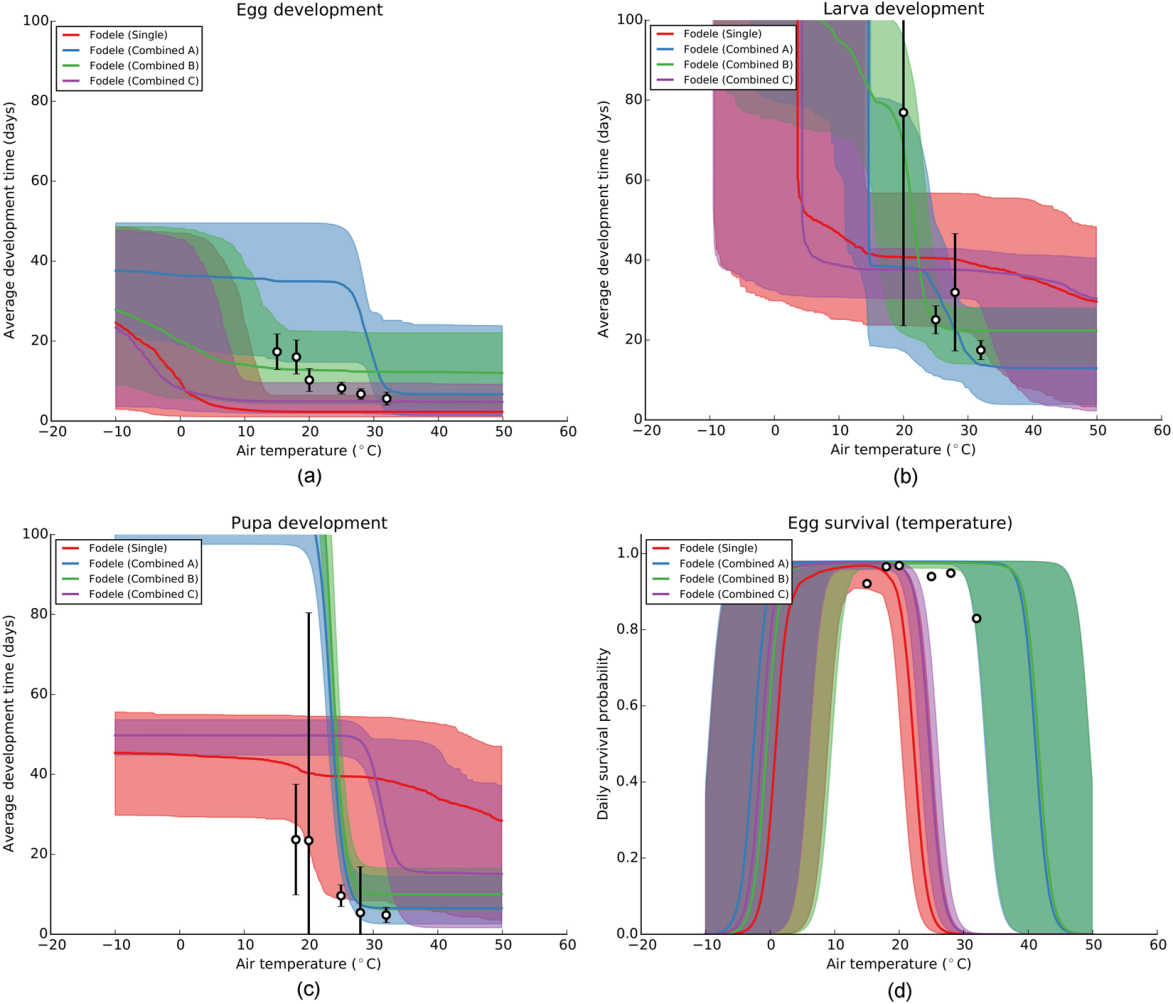
Representative functional forms of environmental dependence inferred for Fodele. In (**a**–**c**), temperature-dependent development times of eggs, larvae, and pupae, respectively, are plotted in units of days. In (**d**), temperature-dependent daily egg survival probabilities are plotted. Solid lines indicate the median and the shaded areas indicate the 95% range of the values predicted by a posterior mode. Circles and vertical lines represent the average and the standard deviation, respectively, of the experimental data (see sec. Experimental datasets).

In this context, we constrained environmental dependence with model structure and longitudinal surveillance data. Although not included in the likelihood nor considered as a prior probability, we assessed whether laboratory-driven life history parameters can be inferred correctly for *Ph*. *papatasi*. In Table 4, we display the average matching scores (${\rm{\Pi }}(\theta )$) and their standard deviations for each posterior mode. In Supplementary Fig. S2, we plot all functional forms of environmental dependence included in the model, and in Fig. 6, four discerning dependences inferred for Fodele where the posterior modes are the least dispersed.

**Table 4:** Inference of environmental dependence using longitudinal surveillance data.

Type	Area	Π(*θ*)
Single	Geri	77596.15 ± 134275.64
Single	Steni	12162.72 ± 3026.91
Single	Fodele	16415.80 ± 2494.93
Combined A	Geri	1366.70 ± 211.05
	Steni	2342.83 ± 468.60
	Fodele	1871.73 ± 341.43
Combined B	Geri	1032.60 ± 196.06
	Steni	1333.26 ± 243.98
	Fodele	1011.27 ± 178.32
Combined C	Geri	14050.91 ± 1695.24
	Steni	13706.81 ± 1574.20
	Fodele	14730.04 ± 1722.69

We found that combined inferences that fit each location equally (Combined A and B) closely resemble the environmental dependence determined with laboratory experiments. The analysis indicated that although they yield more constrained posterior modes, larger datasets from single locations do not necessarily yield better predictions of environmental dependence. In addition, the unbalanced fit among multi-location inferences, Combined C, which leans towards Fodele, does not agree well with laboratory data.

**Figure 7:**
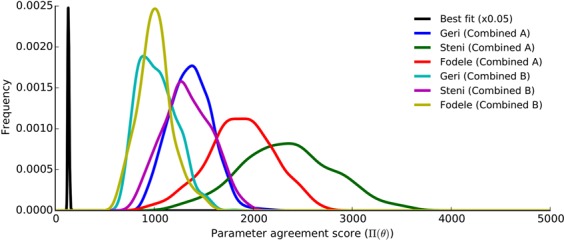
Agreement of Combined A and B with laboratory-derived parameter values. Kernel density estimates of the parameter agreement scores (${\rm{\Pi }}(\theta )$) are plotted for the three versions of Combined A and B along with the best fit, which was obtained by excluding the likelihood. Frequency of the best fit distribution (black) is scaled down by a factor of 0.05 to aid in visualisation.

In the framework of this study, we obtained the best-possible agreement by omitting the likelihood and performing inference only on the prior probability. When we compared this best fit with the agreement scores (${\rm{\Pi }}(\theta )$) of Combined A and B, we found that although Combined A and B demonstrate a close match, they do not overlap with the best fit (Fig. 7). The evident divergence from the maximum agreement indicates that a certain degree of deviation from the experimentally-derived values is needed in order to explain the surveillance data. Accordingly, we argue that direct use of experimental results in mathematical models of population dynamics might be misleading.

**Figure 8:**
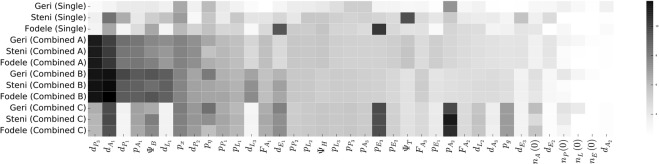
Parameter sensitivity analysis of posterior modes for *Ph*. *papatasi*. Color gradient shows the logarithm of the parameter sensitivities where darker colours indicate higher values. Parameters are sorted from left to right with a descending order with respect to the sum of their sensitivities in posterior modes Combined A and B.

In order to assess the information content of the surveillance data, we performed parameter sensitivity analysis using a multivariate Gaussian approximation around the maximum of each posterior mode^18,40^. We calculated the sensitivity matrix ${\mathcal H}$ as the inverse of the variation of each Gaussian, and normalised with the range of the search domain for each parameter (see Table 1). We report, in Fig. 8, the logarithm of the *n*^*th*^ diagonal element of ${\mathcal H}$ as the sensitivity of the *n*^*th*^ parameter.

As a result, we found that surveillance datasets constrain parameters in different ways and sugest alternative environmental dependences except from Combined A and B. According to Table 4, Combined A and B exhibit the highest resemblance to the laboratory data, therefore, are more likely to suggest biologically plausible environmental dependences.

Our results reveal that, parameters consistently associated with high sensitivity are (i) minimum duration of the gonotropic cycle (${d}_{{A}_{1}}$), (ii) median developmental temperature for pupae (${d}_{{P}_{3}}$), (iii) probability of a female progeny (${p}_{x}$), (iv) breeding site habitability (${{\rm{\Psi }}}_{B}$), and (v) capture probability (${p}_{\eta }$). In addition, for Combined A and B, sensitivity is also high for minimum mean development time of pupae (${d}_{{P}_{1}}$) and larvae (${d}_{{L}_{1}}$), and maximum daily survival probability of adults (${p}_{{A}_{1}}$). On the other hand, the relative humidity threshold (${{\rm{\Psi }}}_{H}$), the temperature threshold for dormancy (${{\rm{\Psi }}}_{T}$), and initial conditions do not have a high impact on model dynamics.

High sensitivity is an *a priori* expectation for ${p}_{x}$, as it is strongly informed by the presence of both female and male sand fly counts in surveillance datasets. High sensitivity was also expected for ${{\rm{\Psi }}}_{B}$, being directly linked with multiple biological processes such as larva and pupa development, and fecundity. Lastly, high sensitivity for ${p}_{\eta }$ was expected due to the direct effect on the observed number of sand flies.

We note that the sensitivity measure we employed depends largely on the characterisation of the posterior distribution. In this context, sensitivity is a measure of impact of a parameter on the dynamics of male and female sand flies. Therefore, the low impact parameters, such as temperature and relative humidity threshold for dormancy, and initial conditions are relatively less potent in determining adult sand fly dynamics according to most of the posterior modes. On the other hand, parameters controlling pupa development and adult survival have high sensitivities due to being directly related to adult population size. An interesting finding is the high sensitivity of the gonotrophic cycle duration that is likely due to the negative effect of egg laying on the survival of adult sand flies.

### Multi-location inferences yield predictive models

We tested model predictability over the surveillance dataset from Adana. The test set contains sightings of *Ph*. *papatasi*, *Ph*. *tobbi*, and the *Ph*. *major s*.*l*. species complex (corresponding to either *Ph*. *neglectus* or *Ph*. *syriacus* in Adana) for the two consecutive years 2011–2012 (see sec. Surveillance and environmental datasets). As a result, we found that the single-location inferences and Combined C do not yield useful predictions for any of the species (see Supplementary Fig. S3). The majority of model predictions using these posterior modes comprise very few or no comparable trajectories with the observations (Table 5). Inclusion of laboratory data as a prior probability for single-location inferences elevated the success rate only for the case of *Ph*. *papatasi* in Steni (raised to $\mathrm{30.7 \% }$ from $\mathrm{0.6 \% }$). Despite this, the fit did not improve considerably (attenuated to 2301.96 ± 2242.85 from 1252.01 ± 153.29).

**Table 5:** Model validation over the test set from Adana.

Species	Type	Area	*ρ*	${\mathscr{S}}$(*δ*|*θ*)
*Ph*. *major s*.*l*.	Single	Fodele	0	—
*Ph*. *tobbi*	Single	Steni	17.5%	1358.76 ± 1273.42
*Ph*. *papatasi*	Single	Geri	0	—
	Single	Steni	0.6%	1252.01 ± 153.29
	Single	Fodele	0.7%	4423.84 ± 161.76
	Combined A	Geri	0.2%	2221.41 ± 124.49
		Steni	99.3%	12522.04 ± 12859.06
		Fodele	96.6%	744.00 ± 478.24
	Combined B	Geri	0	—
		Steni	91.9%	5654.38 ± 7651.19
		Fodele	62.4%	1250.66 ± 740.88
	Combined C	Geri	0	—
		Steni	0	—
		Fodele	0	—

We note that the test set does not contain many observations of *Ph*. *major s*.*l*., which led us to assume that low abundance may indicate low suitability for the establishment of any species in the complex including *Ph*. *neglectus*. In turn, the model may correctly predict low suitability. Although the success rate for *Ph*. *tobbi* is low, we found that the successful trajectories (17.5%) agree well with the observations. We note that *Ph*. *neglectus* and *Ph*. *tobbi* were observed each in only one location in the training set; therefore, more data from alternative locations are needed to draw reliable predictions for these species.

**Figure 9:**
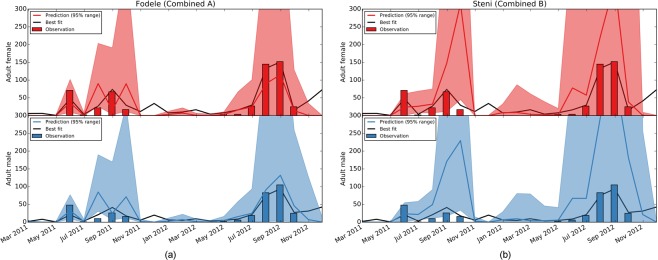
Model validation over Adana, Turkey. Median (solid lines — red and blue) and the 95% range (shaded regions) of predictions using Combined A for Fodele (left) and Combined B for Steni (right) over the test set. The black lines indicate the median trajectories (black lines) obtained using the optimum posterior mode inferred for the region.

We found that Combined A and B — the multi-location inferences that demonstrate a balanced fit to each location — successfully predict *Ph*. *papatasi* abundance (Fig. 9). We obtained high variability in the predictions due to the intrinsic stochasticity of the model and the uncertainty in parameter values. Despite this, we calculated high success rates. We followed the single-location inference procedure to identify an optimum posterior mode for the *Ph*. *papatasi* in the test set. We found that the median trajectories with Combined A and B are close to the median of the optimum posterior mode endorsing the reliability of the predictions.

**Figure 10:**
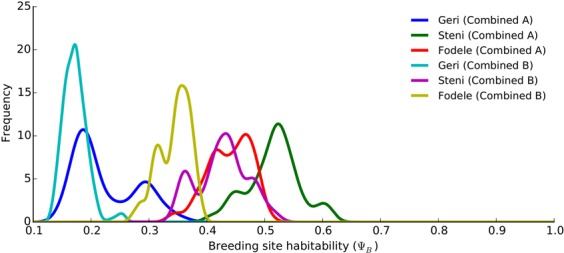
Marginal distributions of breeding site habitability, ${{\rm{\Psi }}}_{B}$, in different variations of Combined A and B. Kernel density estimates of the marginal distributions of ${{\rm{\Psi }}}_{B}$ are plotted for Combined A and B in Geri, Steni, and Fodele.

We note that Combined A and B each have different variations for the three locations in the training set. It appears that the main difference between each variation is the overall vector abundance, which is mainly controlled by breeding site habitability, ${{\rm{\Psi }}}_{B}$ (Fig. 10). Despite the differences between Combined A and B in the remaining parameters, we observed that the variations with ${{\rm{\Psi }}}_{B}$ between 3.5 and 4.5 explain better the observed abundance in the test set.

## Conclusion

Comprehensive studies concerning the environmental dependence of vector species require considerable effort and the availability of suitable rearing facilities^41^. For this reason, vital information required for predictive modelling is scarce for many important vectors. It is clear that flexible strategies exploiting already available or relatively easily obtainable data are needed. Vector surveillance, which is usually performed by authorities for monitoring and control, provides a viable source of information.

Here, we demonstrate that predictive models can be constructed by using field observations, while laboratory studies inform model structure and impose known biological constraints. The model we presented is structurally informed by the known biological mechanism and the environment-dependence of well-studied sand fly species. Parameters of the model are configured to represent a specific vector species by exploiting various amounts of surveillance data.

Our analyses corroborate that in order to construct predictive models, surveillance data collected from multiple independent sites are needed. In addition, a posterior sample of parameters should demonstrate a balanced fit by representing each site adequately to yield a predictive model. As a result, we observed that *Ph*. *papatasi* dynamics in Adana, Turkey, can be predicted with parameters inferred using surveillance data from Crete and Cyprus.

We note that the surveillance areas considered in this study share many environmental characteristics. Although our results suggest that the model may be applicable to other regions with similar characteristics, large-scale regional and global applications should take account of diverse surveillance datasets comprising observations from different environmental conditions.

Our results suggest that the most predictive parameter configurations are also informative about the environmental dependence of *Ph*. *papatasi*. Although laboratory-derived life-history parameters were not explicitly imposed in the form of prior probability, the inferred parameter values agree well with the experimentally-determined values. Regardless of the agreement, we obtained large variations especially in the lower and upper bounds of temperature dependence. This is indicative of the absence of observations during such extreme conditions, which can be compensated by constraining the corresponding parameters explicitly with regards to expert knowledge.

We note that humidity, despite being indicated as a key environmental dependence^11,24,25^, was not informed adequately by the surveillance data — only a substantially low threshold was identified as critical. It is possible that a more biologically-tailored model structure and more detailed observational datasets are needed to provide the required resolution to discern humidity dependence. We suggest the inclusion of day-and-night temperature and humidity differences as a possible next step since such data were reported to correlate strongly with vector abundance^42^. We also note the possibility of the existence of a posterior mode with a higher probability and predictive capacity than the ones identified in this study. Further improvement of the model and the identification of predictive models for *Ph*. *neglectus* and *Ph*. *tobbi* will be the subject of future research.

An additional factor contributing to the variance between observations and predictions is the assumed range of applicability of our model. The distance between collection sites and the closest available meteorological stations is a limiting factor. Furthermore, it is not trivial to include microclimatic conditions due to the difficulty of obtaining or predicting such information. At present, the most detailed global climate simulations are provided at 0.25 degree resolution, which corresponds to about 25 km near the equator^43^. With the improvement of global and regional climate models and the public availability of such data, we expect that the accuracy of vector and disease dynamics predictions will improve considerably.

It is important to note that none of the inferred parameter values, despite some demonstrating high predictive ability, provides the maximum possible fit to the laboratory-derived values. This indicates that extra care is needed when applying laboratory data to a larger scale. Experiments reflect vector biology under controlled environmental conditions. The scale of such studies may be related, to some extent, to the microclimatic environment around breeding sites. The scale of a typical field study, however, is several square kilometers at best. The methodology we presented offers a potential solution to this problem by allowing environmental dependence to be informed, and therefore adjusted to data collected in the filed.

## Supplementary information

Supplementary Figures S1 S2 S3

Supplementary file S1

## Data Availability

All data generated or analysed during this study are included in this published article (and its Supplementary Information files).
