# Supplementary material for: A climate-driven and field data-assimilated population dynamics model of sand flies
Source: Sci Rep. 2019 Feb 21;9:2469. doi: 10.1038/s41598-019-38994-w (PMC6385250; doi:10.1038/s41598-019-38994-w)
Supplement: Supplementary file 1 — Supplementary Figures S1 S2 S3 [file 41598_2019_38994_MOESM1_ESM.pdf]

A climate-driven and field data-assimilated population dynamics model of sand flies

Kamil Erguler, Irene Pontiki, George Zittis, Yiannis Proestos, Vasiliki Christodoulou, Nikolaos Tsirigotakis, Maria Antoniou, Ozge Erisoz Kasap, Bulent Alten, Jos Lelieveld

Supplementary Figure S1

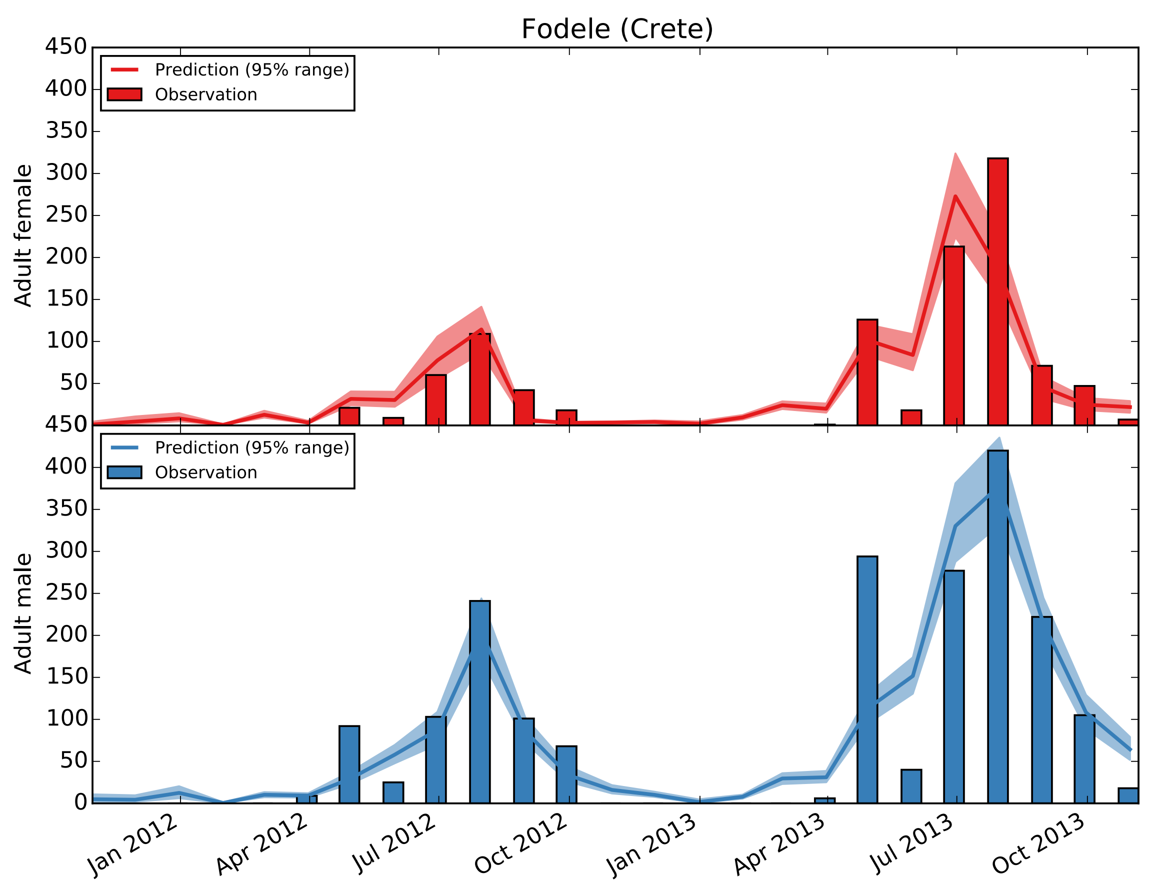

a) Single-location inference for *Ph. neglectus*

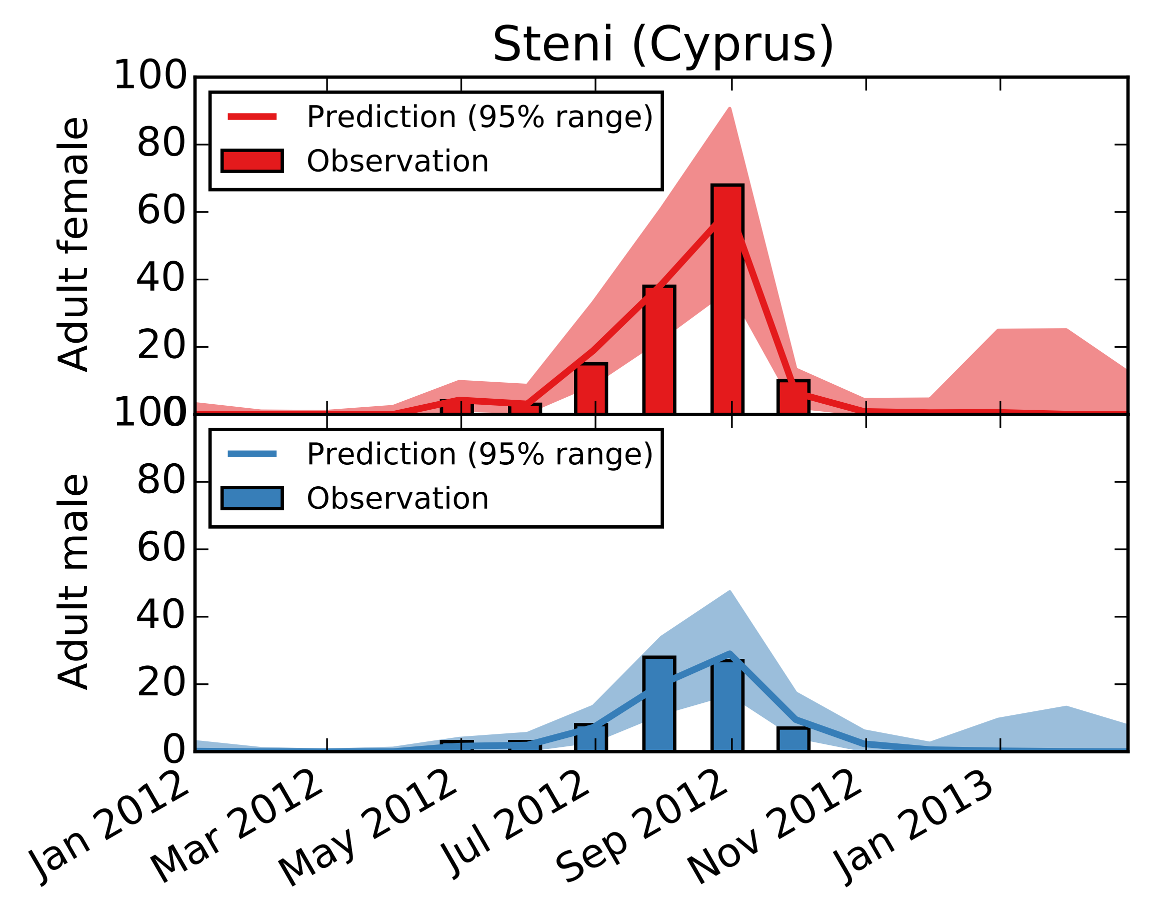

b) Single-location inference for *Ph. tobbi*

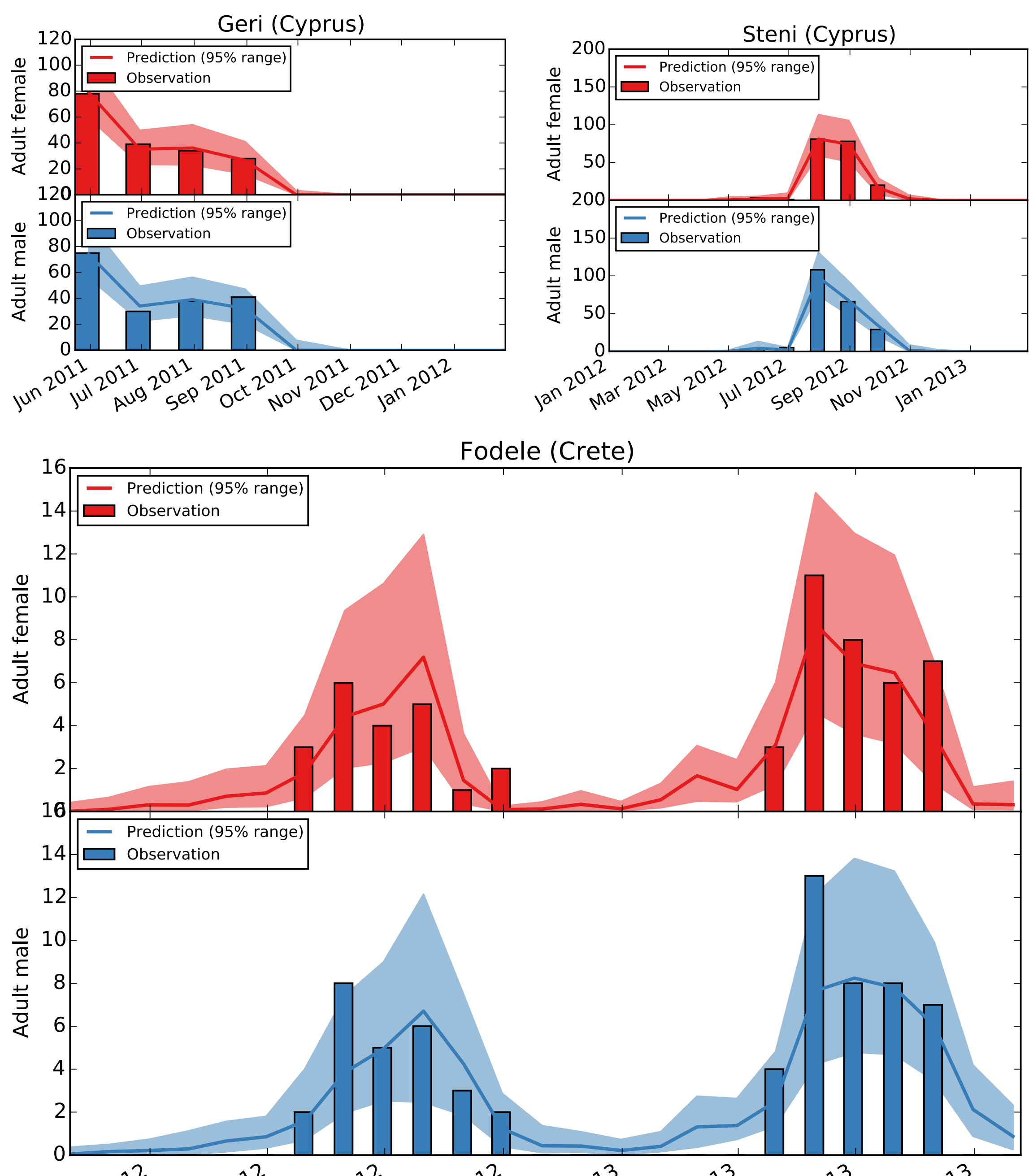

c) Single-location inferences for *Ph. papatasi*

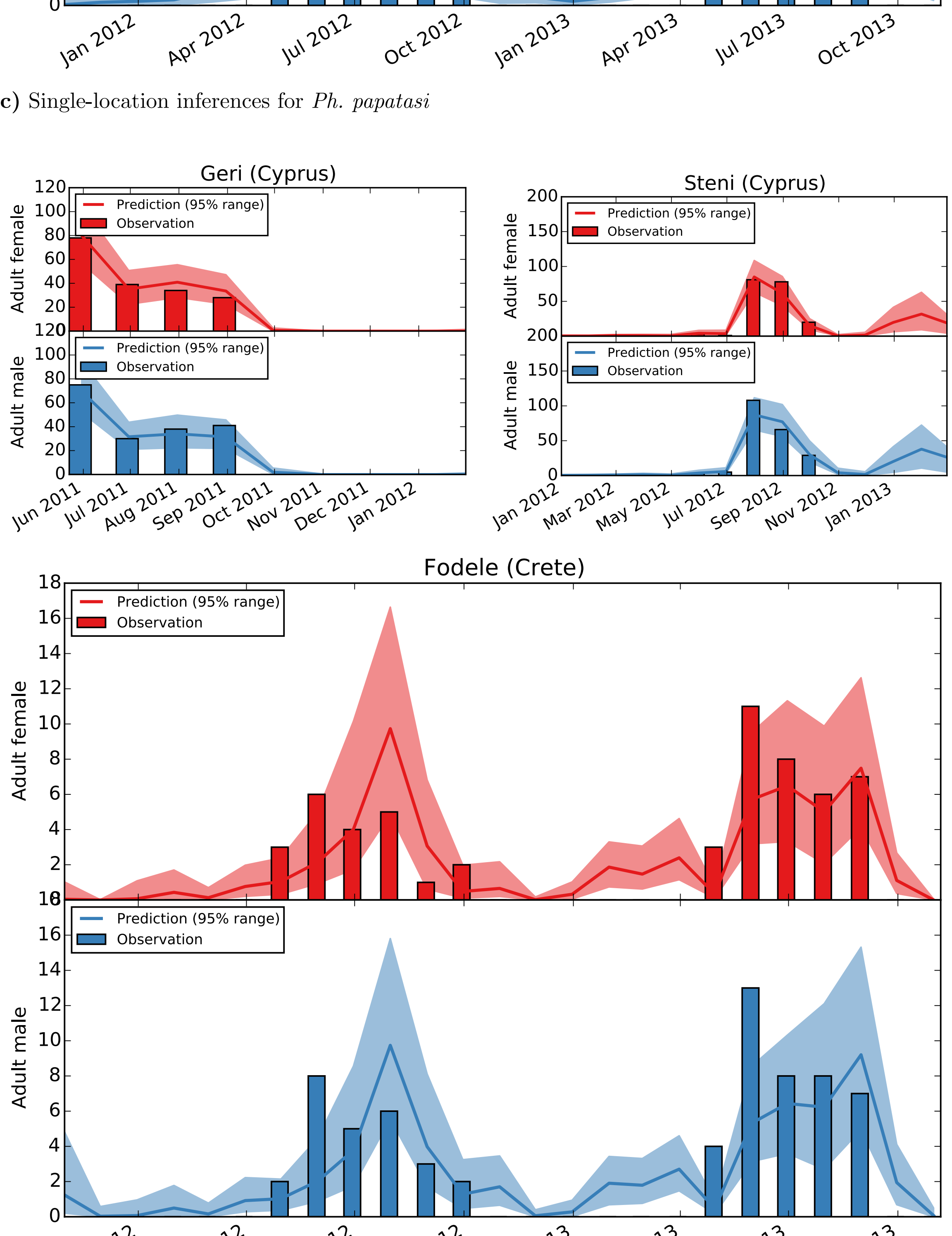

d) Combined A for *Ph. papatasi*

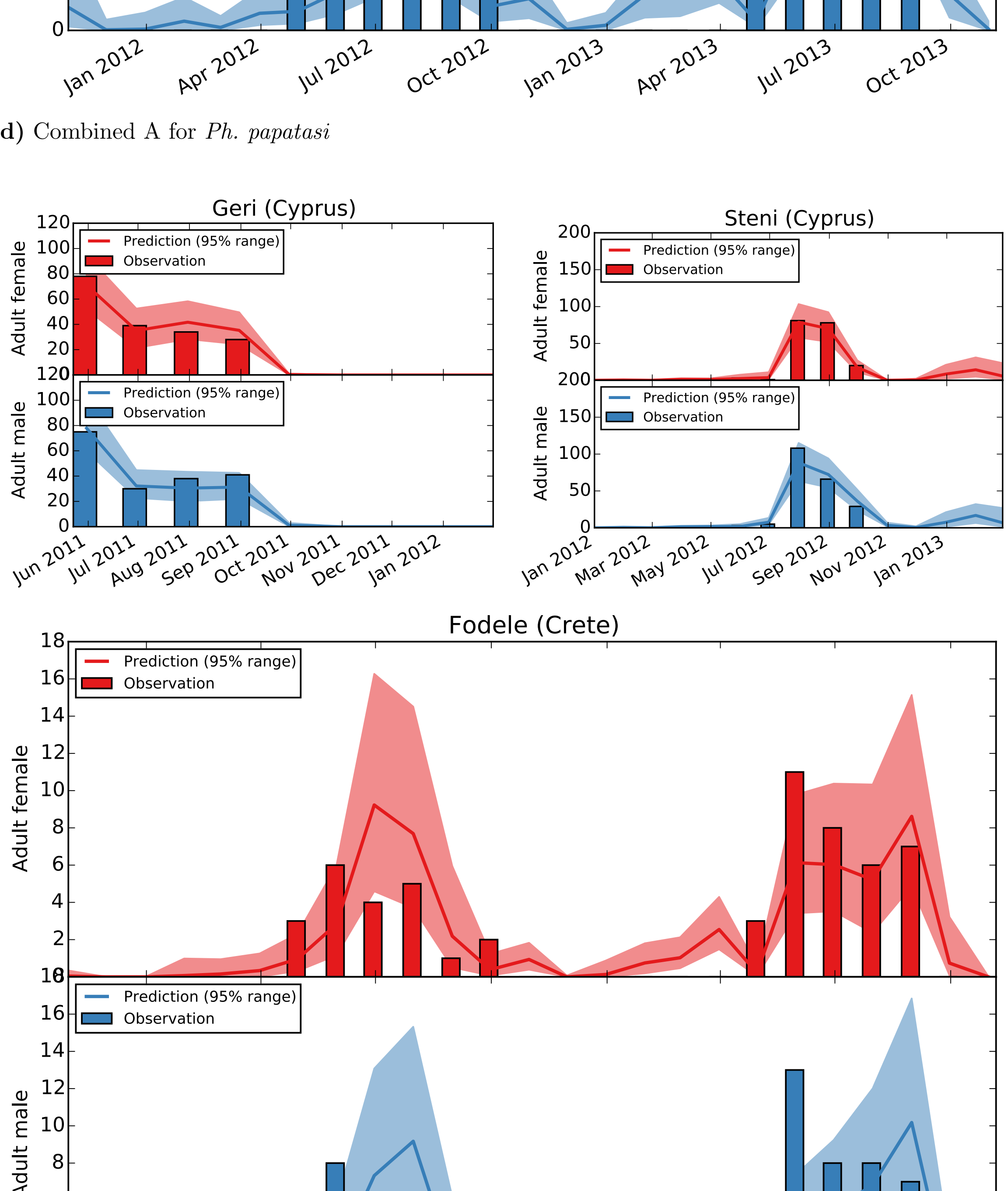

e) Combined B for *Ph. papatasi*

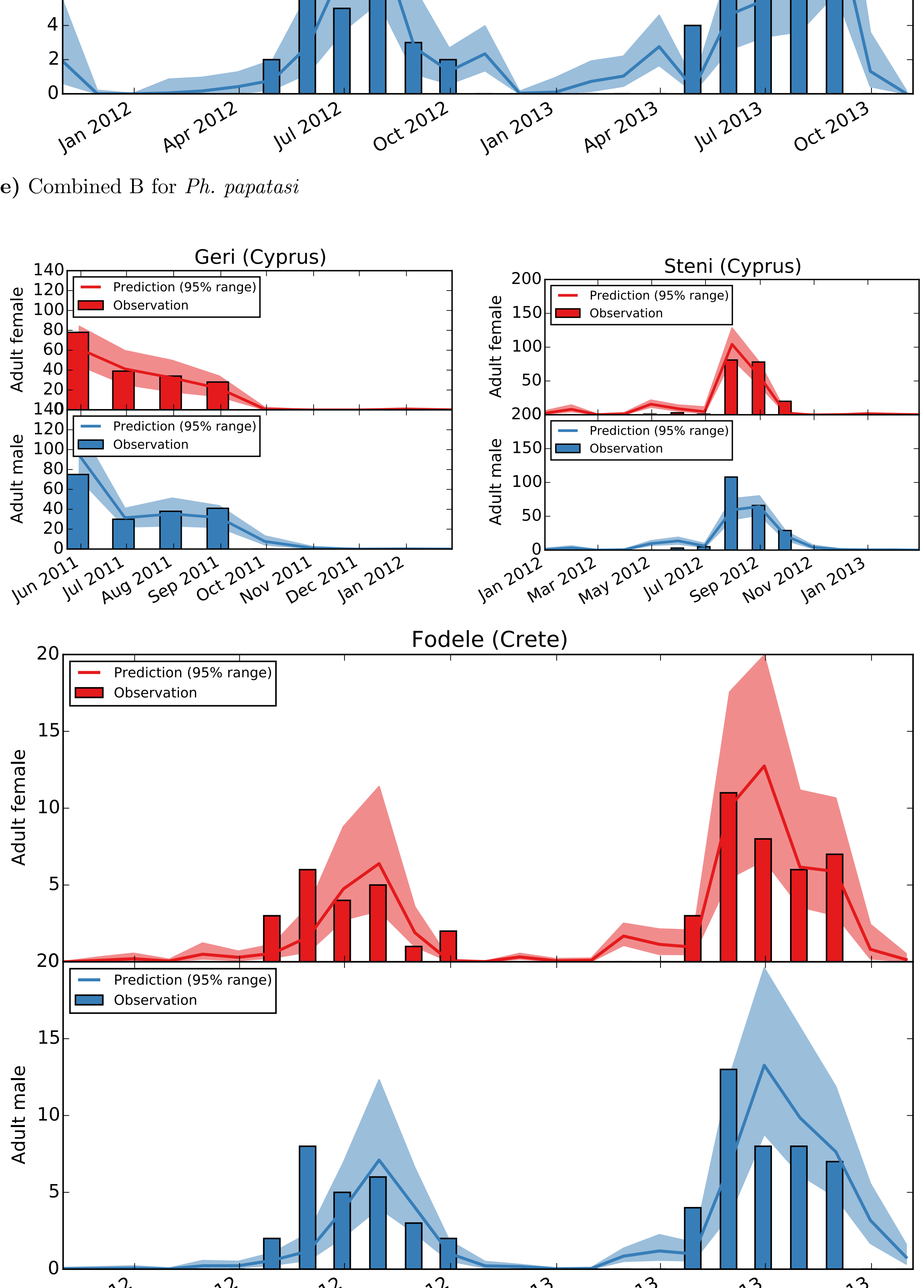

f) Combined C for *Ph. papatasi*

Figure S.1. Agreement between model predictions and the observations in the training set. Simulations from inferences performed using a single location are presented in (a), (b), and (c) for *Ph. neglectus*, *Ph. tobbi*, and *Ph. papatasi*, respectively. Simulations from combined inferences A, B, and C are plotted in (d), (e), and (f), respectively, for *Ph. papatasi*. Data and predictions for female (red) and male (blue) adult sand flies are given separately. Observations are shown with bars, which correspond to the total number of collections at the location for a given time point. Median and 95% range of predictions are plotted with a solid line and a shaded area, respectively.

A climate-driven and field data-assimilated population dynamics model of sand flies

Kamil Erguler, Irene Pontiki, George Zittis, Yiannis Proestos, Vasiliki Christodoulou, Nikolaos Tsirigotakis, Maria Antoniou, Ozge Erisoz Kasap, Bulent Alten, Jos Lelieveld

Supplementary Figure S2

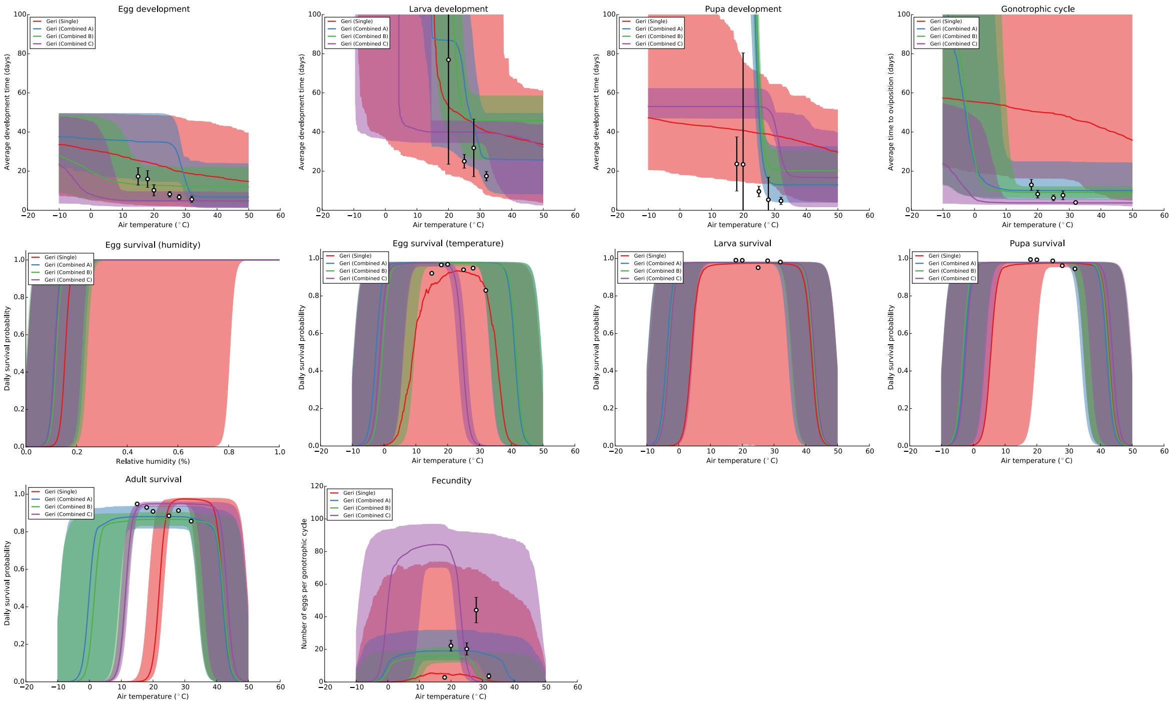

a) Inference of environmental dependence for Geri (Cyprus)

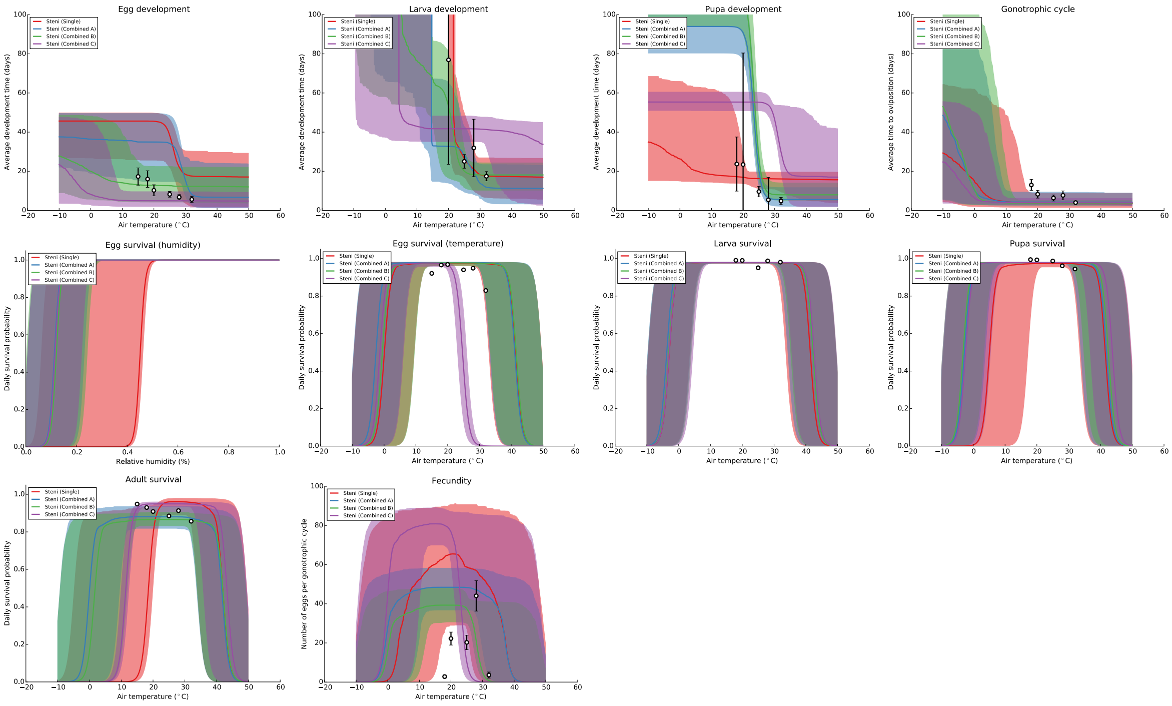

b) Inference of environmental dependence for Steni (Cyprus)

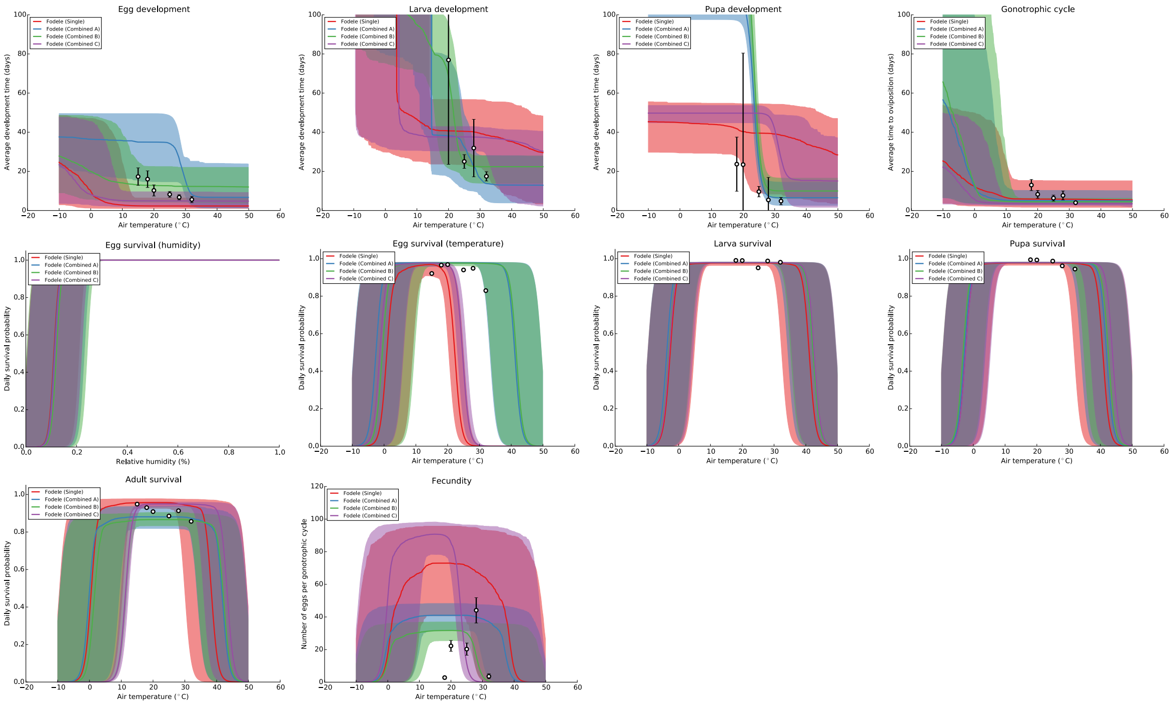

c) Inference of environmental dependence for Fodele (Crete)

Figure S.2. Functional forms of environmental dependence. Solid lines and the shaded areas indicate the median and the 95% range. Circles and vertical lines represent the average and the standard deviation, respectively, of the experimental data.

Supplementary Figure S3

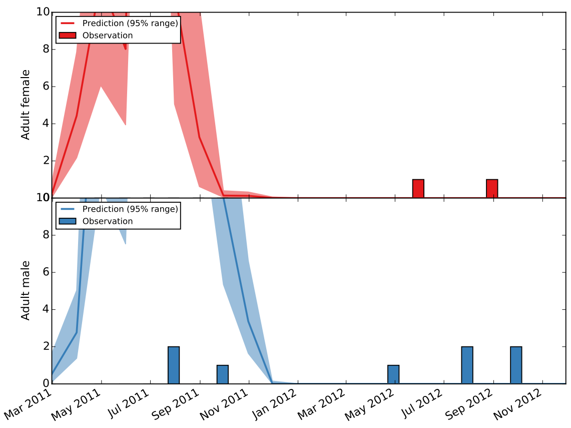

a) Single-location inference for *Ph. major s.l.*

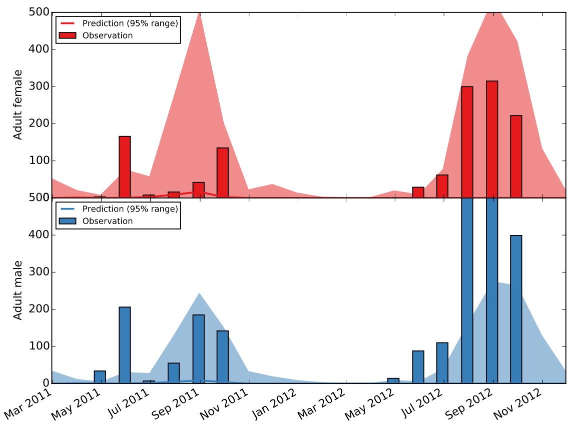

b) Single-location inference for *Ph. tobbi*

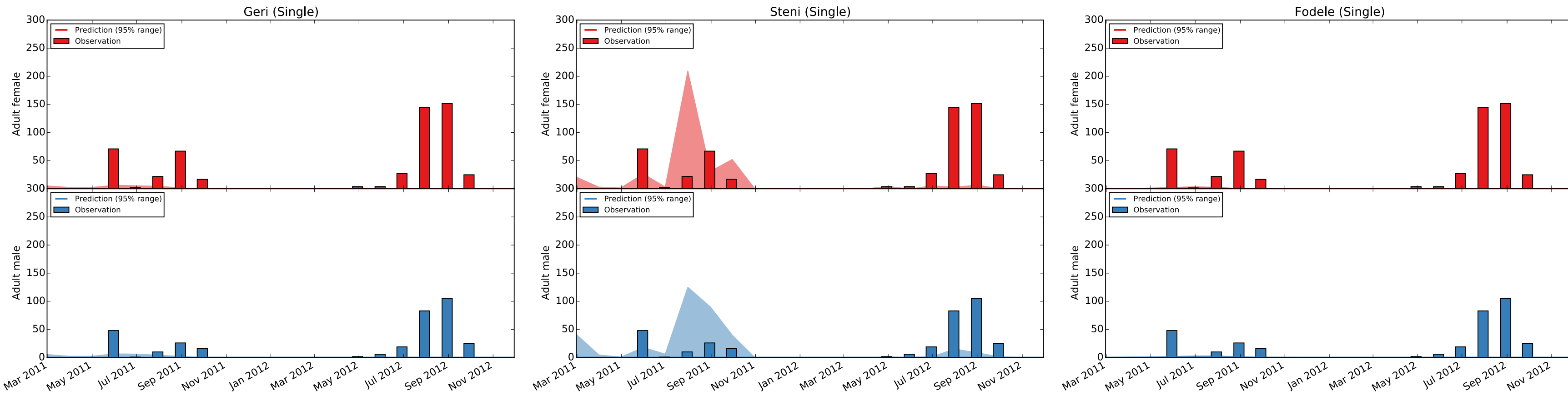

c) Single-location inferences for *Ph. papatasi*

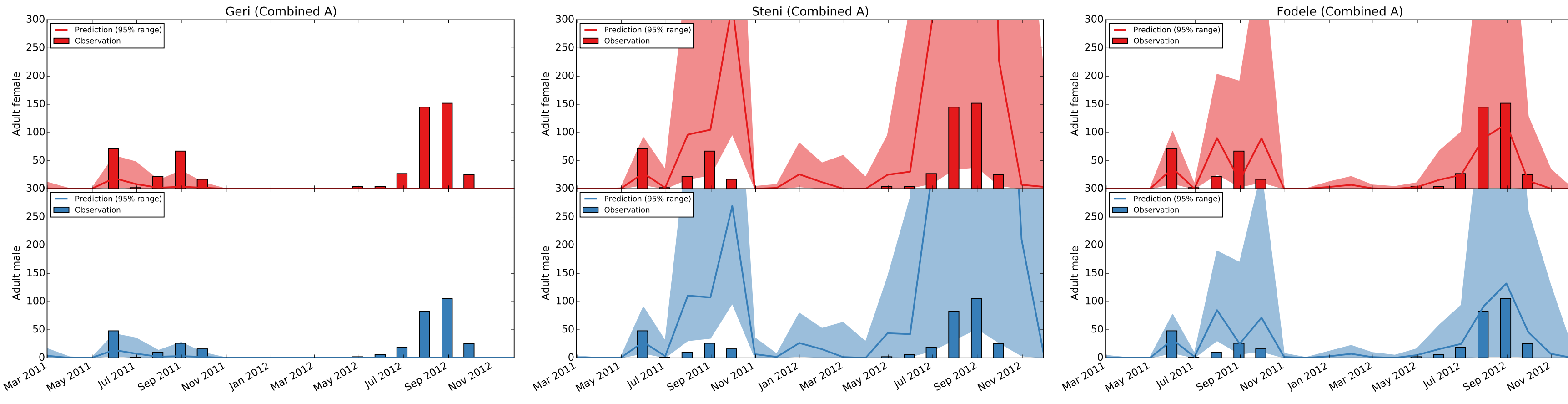

d) Combined A for *Ph. papatasi*

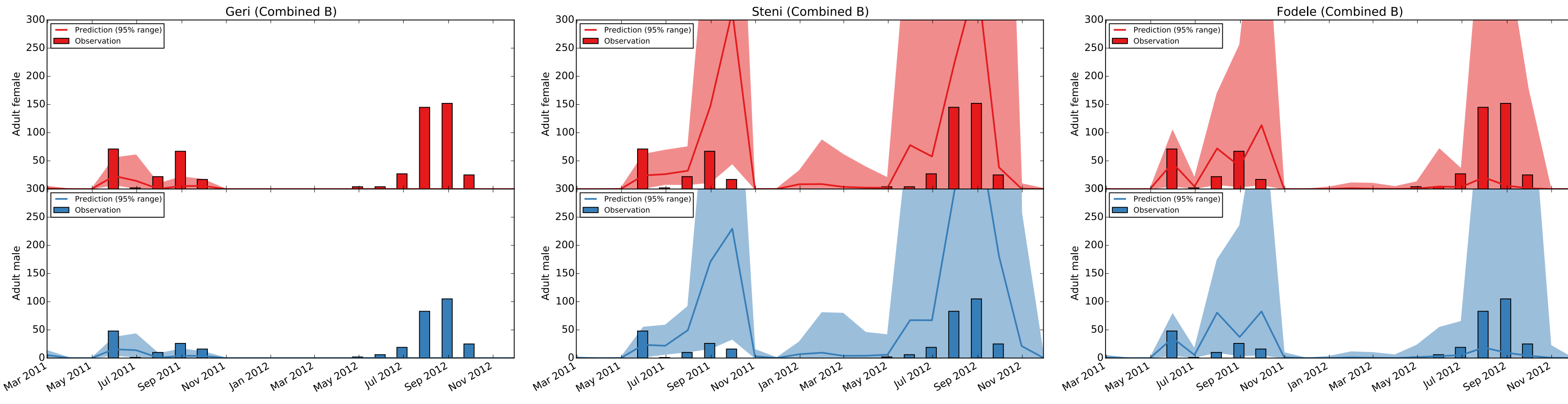

e) Combined B for *Ph. papatasi*

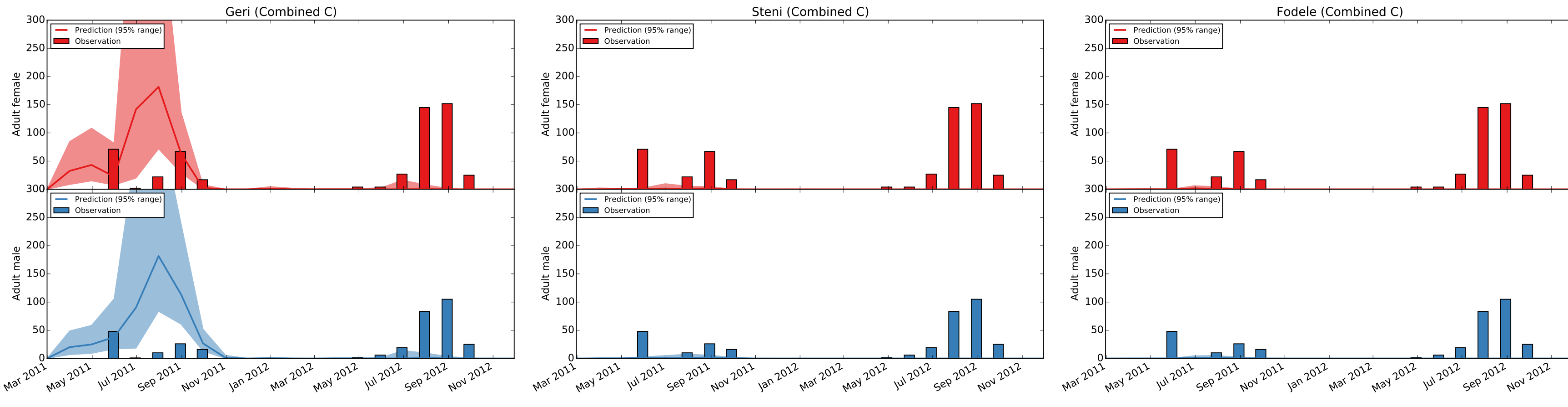

f) Combined C for *Ph. papatasi*

Figure S.3. Model performance over the test set with different posterior modes. Prediction of *Ph. major s.l.* (a), *Ph. tobbi* (b), and *Ph. papatasi* (c-f) abundance in the Koyunevi region of Adana. Predictions were performed using single-location inferences for *Ph. major s.l.* and *Ph. tobbi*. For *Ph major s.l.*, the single-location inference for *Ph. neglectus* was used. For *Ph. papatasi*, predictions are displayed both with the three single-location inferences and with the three multi-location inferences (Combined A-C).
